# Night work, mortality, and the link to occupational group and sex

**DOI:** 10.5271/sjweh.3892

**Published:** 2020-09-01

**Authors:** Torbjörn Åkerstedt, Jurgita Narusyte, Pia Svedberg

**Affiliations:** 1 Division of Psychology, Department of Clinical Neuroscience, Karolinska Institutet, Stockholm, Sweden; 2 Stress Research Institute, Stockholm University, Stockholm, Sweden; 3 Division of Insurance Medicine, Department of Clinical Neuroscience, Karolinska Institutet, Stockholm, Sweden

**Keywords:** blue-collar work, cancer, cardiovascular, exposure, gender, night shift, occupation, shift work, shift worker, twin, white-collar work

## Abstract

**Objective:**

Night shifts are associated with several major diseases. Mortality has been studied only to a limited extent, and the association with night shifts remains unclear. The purpose of the present study was to investigate the association between duration of night shift exposure and mortality in a large sample from the Swedish Twin Registry (the SALT cohort).

**Methods:**

Cox proportional hazards regression models were used to analyze the data (N=42 731) over a follow-up period of 18 years, with years of night shift work as the exposure variable and adjustment for lifestyle factors and age, and stratification on gender and occupational group.

**Results:**

The hazard ratio (HR) for “ever” night shifts for total mortality was 1.07 [95% confidence interval (CI) 1.01–1.15] but 1.15 (95% CI 1.07–1.25) for longer exposure (>5 years). Also, HR for cause-specific mortality due to cardiovascular disease was significant, with higher HR for longer night shift exposure. Mortality due to cancer was significant for longer exposure only. White-collar workers showed significant HR for longer exposure. In particular, male white-collar workers showed a significant HR, with a highest value for longer exposure [HR 1.28 (95% CI 1.09–1.49)]. Heredity did not influence the results significantly.

**Conclusions:**

Long duration of exposure to night shift work is associated with increased mortality, particularly in male white-collar workers. The lack of effects of accumulated exposure suggests that the results should be interpreted with caution.

Shift work affects 19.8% of the employed population in Sweden (1). Furthermore, shift work that involves night shifts is associated with a higher risk of ischemic heart disease (2), diabetes (3, 4), accidents (5) and breast cancer (6). Recent studies also found that exposure at young age to night work is related to multiple sclerosis (7) and to rheumatic arthritis (8).

Logically, one would expect the increased risk of disease to be associated also with mortality, at least if the risk of the disease is high and the disease represents a large part of the diseases leading to death. The evidence here is weak, however, and the available studies are few in contrast to the number of studies focusing on diseases, sleep, fatigue, or accident risk. This may be due to difficulties in linking night work exposure to mortality on a national level across occupations. Thiis-Evensen (9) was the first to address the issue of shift work and mortality, but he did not find any difference in mortality in a group of 498 shift versus 212 day workers of a Norwegian company over 31 years of study. Taylor & Pocock (10) studied 8603 workers in England and Wales, finding no excess risk of mortality in shift workers, although a reanalysis of these data concluded that there was an increased risk (11). Karlsson et al (12) also failed to find an association among male paper mill workers, but the study was small and women were not included. But Gu et al (13) found an increased risk of mortality among 78 000 female nurses in the US with rotating shifts (ie, with night work), as did Jörgensen et al (14) among 18 000 Danish nurses.

The studies above investigated particular occupational groups or regions. Among studies of population-based representative samples, an increased mortality risk was demonstrated in female white-collar night shift workers (15), but number of years of exposure were not available (N=20 000). Nätti et al (16) investigated 4500 individuals in a representative sample of the Finnish population and found a significant doubling of mortality, but only 190 night workers participated. However, a recent meta-analysis including three of the studies listed above did not find a significant effect of night shifts on mortality (17). Furthermore, a study of a representative sample of 159 000 individuals in Denmark did not find a significant association between shift work and mortality (18), but that study did not adjust for lifestyle variables.

A meta-analysis of 5249 men in various occupations (19) did not find a difference in cardiovascular mortality between day and shift work (all types combined, ie, also those without night shifts).

All cited studies above defined exposure at baseline and did not consider years of exposure, which should be an important factor. Only the studies by Akerstedt et al (15), Nätti et al (16), and Hannerz et al (18) used representative samples of the population, and there is a risk that data from particular occupational groups may not be representative of the population. In addition, most studies, thus far, have been quite small. Furthermore, the standard approach in studies of shift work and health is to compare shift to non-shift workers. This leaves room for selection biases with regard to education, physical workload, work environment, socio-economic group and other factors. These are, to a large extent, variables that differ between white- and blue-collar workers (WCW and BCW). It seems reasonable to separate the latter two groups when studying effects of night work on mortality. There is also a possibility that the link between night work and mortality may be influenced by familial (genetic and shared environmental) factors. This has never been addressed before, and the fact that at least diurnal type is a heritable trait (20) makes an association plausible. Also, mortality has shown to be influenced by genetics (21). A twin approach will make it possible to take shared family environmental and genetic factors into account when investigating the association between night work and mortality.

There seems to be a need for a study on night shifts and mortality using a reasonably large representative sample with limited occupational bias and accumulated exposure to night shifts as a key variable. Thus, a relatively old cohort was used to obtain individuals with close to maximum exposure when the study started. The aim of the present study was to investigate the association between night work (with information on the duration of exposure) and mortality, using data from the Swedish Twin Registry. A special focus was on occupation, gender, and the influence of familial factors on the association. Furthermore, cause-specific mortality in cardiovascular disease and cancer was included.

## Method

### Design

The design was a prospective cohort study. For this study, twins born in Sweden 1900–1958 and who participated in the Screening Across the Lifespan Twin (SALT) study, conducted by the Swedish Twin Registry, were included. Ages at the time of the interview were 41–99 years. Each individual participated in the SALT computer-assisted, telephone interview once between 1998 and March 2003. The response rate was 74% and the total sample encompassed 42 731 individuals with responses on history of night work. Of these, 12 850 had been exposed to night work. The interview included a number of items regarding different diseases, symptoms, lifestyle, night shifts and sociodemographic factors. The procedure for data collection has previously been described in detail elsewhere (22). Data on death were obtained from the Cause of Death Registry at the National Board of Health and Welfare. Data were linked to the twins by using the unique person identification number available for all Swedish citizens.

### Variables

The exposure to night shifts was defined based on the question: “For how many years have you had work hours that included night shifts at least once per month”. The exposed group was defined as participants with 1–45 years of night work. This group was further categorized as short (1–5 years) or longer (6–45 years) exposure, since it seemed reasonable to expect the effect of exposure not to be immediate. The selected intervals are compromises between a balanced number of participants and what was considered to be a short-term exposure. For completeness, we also tried one subdivision of 1–5, 6–10, 11–20, and 21–45 years of night shift work for a sensitivity analysis, as well as another subdivision of 1–14 and 15–45 years. The reference group contained those who reported 0 years of night work (“never night work”).

The outcome variable was defined as total mortality due to any cause of death. For cause-specific analyses, the main diagnosis behind the cause of death was obtained from the Cause of Death Registry. The mortality was analyzed separately due to cancer (ICD-10: C00-D48), circulatory (ICD-10: I00-I99) diseases. In cause-specific mortality analyses, death cases due to those other than the analyzed one were excluded.

The following covariates were included: educational level (0=more than compulsory [reference], 1=compulsory); tobacco use (0=no tobacco [reference], 1=tobacco use (includes current or previous regular smoking/snuffing as well as occasional smoking or snuffing)); alcohol consumption (0=no alcohol [reference], 1=alcohol consumption); leisure-time physical activity (0=moderate exercise [reference], 1=low exercise, 2=high exercise); body mass index (BMI) (0=normal weight (>18.5–25 kg/m^2^) [reference], 1=underweight (≤18.5 kg/m^2^), 3=overweight (>25–30 kg/m^2^), 4=obesity (>30 kg/m^2^)); coffee consumption (0=no coffee [reference], 1–2 cups a day, 3–4 cups a day; >4 cups a day). Severity of disease was assessed by asking SALT participants “Do you have or have you had [here 53 different health problems and conditions were mentioned]” and responses were classified, based on their most severe illness, into categories: 0=no disease, 1=not at all life-threatening (eg, migraine), 2=somewhat life-threatening (such as high blood pressure), and 3=life threatening (eg, stroke, cancer, ischemic heart disease), according to the expected impact of the disease. A more detailed description of this variable and categorization is found elsewhere (23, 24). In addition, self-reported interview data on occupation were coded into occupational groups according to Statistics Sweden (SSYK 96) that were used both as covariates and for stratification. The occupations were grouped into WCW [for example, managers, professionals, office workers, technicians, officers, nurses (codes: 1-4, 011, 021)] and BCW including occupation areas [for example, industrial workers, craftsmen, nurses assistants, drivers, miners, etc (codes 5–9, 031)] according to the criteria of Statistics Sweden.

### Statistical analyses

Frequencies were used to describe the background and covariates for groups with 0 and 1–45 years of night work, and for both groups combined. The difference between occupational categories were tested using Chi^2^ analysis. Exposure was defined as number of years with night shifts. All individuals contributed with time in years until the date of death or the end of follow-up (ie, 31 December 2014). Multiple Cox proportional hazard regression analyses were used to compute hazards ratios (HR) with 95% confidence intervals (CI). After analysis of interaction with exposure, stratification was carried out for BCW and WCW and gender (males/females) across BCW and WCW groups. The analyses were adjusted for within-twin pair dependency by clustering on twin identity. The association was also adjusted for the covariates that were entered stepwise. Only individuals with complete data were included in the analyses of associations. We also made a second analysis with all individuals included.

To assess the influence of familial factors (ie, genetics and family environment), a conditional Cox proportional hazard regression analysis was carried out of same-sex twin pairs that were discordant (different) on mortality. These analyses are based on the fact that twins in a pair are optimally matched on genetics [100% for monozygotic (MZ) or 50% for dizygotic (DZ) twins] and shared (early family) environment (100% for both MZ and DZ twins) as well as age and sex. The familial factors are suggested to be of importance for the studied association if HR computed in conditional (discordant twin) analyses differ from the HR computed in the total sample. Further, a difference between the association estimated for MZ and DZ twins would suggest the importance of genetic factors. All analyses were performed using SAS V.9.4 (SAS Institute, Cary, NC, USA).

### Ethical considerations

The Regional ethical committee of Stockholm, Sweden approved this study.

## Results

Descriptive statistics of the sample, including background variables, are presented in table 1, and 30.1% of the sample had worked night shifts. Note that these values represent accumulated night shifts over much of a working career. The table shows a higher prevalence of men, overweight, smoking, blue-collar jobs among night shift workers.

**Table 1:** Descriptive statistics of the sample (N=42 731) stratified by night work.

	All	Night work	
	
	(N=42 731) ^a^	1–45 years (N=12 850) ^a^	0 years (N=29 881) ^a^
	
	N (%)	N (%)	N (%)
Sex			
Women	22 884 (54)	5252 (41)	17 632 (59)
Men	19 847 (46)	7598 (59)	12 249 (41)
Civil status			
Single/widow/separated	11 589 (27)	3488 (27)	8101 (27)
Married/cohabiting	31 135 (73)	9362 (73)	21 773 (73)
Body mass index			
Underweight	571 (8)	119 (1)	452 (2)
Normal	22012 (53)	6079 (48)	15 993 (55)
Overweight	15 740 (38)	5206 (41)	10 534 (36)
Obese	3427 (8)	1253 (10)	2174 (7)
Missing	981	193	788
Education			
Compulsory	20 455 (51)	5977 (50)	14 478 (51)
More than compulsory	19 992 (49)	6087 (50)	13 905 (49)
Missing	2284	786	1498
Smoking			
Yes	11 411 (27)	4142(32)	7269 (24)
No	31 316 (73)	8706 (68)	22 610 (76)
Missing	4	2	2
Coffee (cups/day)			
0	2727 (6)	811 (6)	1916 (6)
1–2	10 663 (25)	2874 (22)	7789 (26)
3–4	16 036 (38)	4397 (34)	11 639 (39)
>4	13 262 (31)	4754 (37)	8508 (29)
Missing	43	14	29
Alcohol consumption			
Abstainers	3837 (10)	1309 (12)	2468 (10)
Seldom drinkers	10 363 (28)	2839 (26)	7388 (28)
Often drinkers	23 241 (62)	6936 (63)	16 043 (62)
Missing	5748	1766	3982
Severity of disease			
No disease	1464 (3)	398 (3)	1066 (4)
Not life-threatening	12 926 (30)	3791 (30)	9135 (31)
Somewhat life-threatening	22 735 (53)	6835 (53)	15 900 (53)
Very life-threatening	5604 (13)	1826 (14)	3778 (13)
Missing	2		2
Leisure-time physical activity			
Low	9677 (25)	2970 (26)	6607 (25)
Moderate	23906 (64)	7271 (63)	16 635 (64)
High	4978 (11)	1296 (11)	2782 (11)
Missing	5170	1313	3857
At work			
Yes (at least half-time or less)	24 234 (80)	7464 (79)	16 770 (81)
No	6034 (20)	2001 (21)	4033 (19)
Missing	12 463	3385	9078
Previous cancer (self-reported)	2995 (7)	843 (7)	2152 (7)
Occupation			
White-collar	20 312 (49)	5295 (42)	15 017 (52)
Blue-collar	21 338 (51)	7426 (58)	13 912 (48)
Missing	1081	129	952
Causes of death during follow-up	9494 (22)	2800 (22)	6694 (22)
Circulatory	3736 (39)	1102 (39)	2634 (39)
Cancer	2983 (31)	892 (32)	2091 (31)
Other	2775 (30)	806 (29)	1969 (29)

a Age (range 41–99): mean 59 [standard deviation (SD)] 11 years.

^b^ Age (range 41–99): mean 58 (SD) 10 years.

^c^ Age (range 41–99): mean 59 (SD) 11 years.

The results of the analyses of the association between night work and mortality are presented in table 2. Night work (1–45 years) was significantly associated with future mortality due to all-cause, as well as circulatory death, after full adjustment. Longer duration (>5 years) showed higher HR and mortality due to cancer disease became significant for longer duration only.

**Table 2:** Cox proportional crude and adjusted hazard ratios (HR) with 95% confidence intervals (CI) for the association between night work and mortality (N=30 582).

Years with night work	N	Model 1 ^a^ HR (95% CI)	Model 2 ^b^ HR (95% CI)
Mortality all causes			
0	21 297	1	1
1–45	9285	1.16 (1.09–1.24)	1.07 (1.01–1.15)
1–5	3987	0.99 (0.90–1.08)	0.97 (0.88–1.07)
6–45	5298	1.30 (1.20–1.40)	1.15 (1.07–1.25)
Mortality due to circulatory disease			
0	19 323	1	1
1–45	8362	1.33 (1.18–1.49)	1.17 (1.04–1.31)
1–5	3646	1.10 (0.93–1.30)	1.05 (0.89–1.24)
6–45	4716	1.51 (1.32–1.73)	1.25 (1.09–1.44)
Mortality due to cancer disease			
0	19 750	1	1
1–45	8463	1.06 (0.96–1.17)	1.05 (0.94–1.16)
1–5	3679	0.86 (0.74–1.00)	0.90 (0.77–1.04)
6–45	4784	1.21 (1.08–1.36)	1.16 (1.03–1.31)

a Crude model.

b Adjusted for sex, age, education, coffee consumption, smoking, body mass index, severity of disease at baseline, white/blue-collar occupational group, alcohol consumption and leisure-time physical activity.

In a sensitivity analysis, we divided years of exposure into groups with 1–5, 6–10, 10–20, and 21–45 years, but no trend in HR values was seen for all-cause mortality. We also tested long exposure as 15–45 years to increase the N for that group (with 1–14 years as short exposure). However, the HR for 15–45 years was 1.13(95% CI 1.03–1.26), compared to HR 1.15 (95% CI 1.07–1.25) for 6–45 years. The results for the intermediate exposures did not change markedly, and the HR were non-significant. Specific causes of mortality were not analyzed because of low number of outcomes.

Since it might be argued that smoking, alcohol consumption, BMI, and leisure-time physical activity could be affected by shift work, we also conducted an sensitivity analysis adjusting for background variables only (age, sex, education, and severity of disease at baseline), and in a next step, also for the variables that could be seen as possibly influenced by shift work. After the adjustment for background variables only, we obtained a HR 1.13 (95% CI 1.06–1.21) for 1–45 years of exposure and HR 1.22 (95% CI 1.13–1.32) for 6–45 years. This should be compared with the second model of table 2 (adjusted for all covariates), which yielded HR 1.07 (95% CI 1.01–1.15) and 1.15 (95% CI 1.06–1.24) for 1–45 years and 6–45 years, respectively. The 1–5-year exposures retained low and non-significant HR and were not affected.

Since effects of night shifts on mortality may attenuate after retirement, we carried out a sensitivity analysis including only those still at work. For 6–45 years of exposure we obtained HR 1.16 (95% CI 1.07–1.27) for those at work (compared to 1.15 (95% CI 1.07–1.25) for the complete sample. The number of participants was 25 892 for those at work and 30 582 for the complete group). For WCW at work with 6–45 years of exposure, we obtained HR 1.28 (95% CI 1.12–1.47) versus HR 1.25 (95% CI 1.09–1.44) for the complete sample (table 2). For BCW, no HR were significant for either group.

Based on the hypothesis of influence of occupation and gender moderation, we also computed the interactions between exposure (0/1–45 years) and occupational group as well as gender. For the former, model 2 showed a P-value of P=0.06 and, for the latter, P=0.01. Since the first was borderline significant, we stratified for both. Stratification for occupational group did not show any significant association for 1–45 years of exposure for either occupational group (although close to significance), but longer exposure showed a significant HR for WCW (table 3). Interaction analysis or exposure with gender showed P=0.07 in the WCW group and P=0.10 in the BCW group.

**Table 3:** Cox proportional crude and adjusted hazard ratios (HR) with 95% confidence intervals (CI) for the association between night work and mortality stratified by occupational groups.

Years with night work	N	Model 1 ^a^ HR (95% CI)	N	Model 2 ^b^ HR (95% CI)
White-collar	16 345			
0	12 099	1	12 099	1
1–45	4246	1.10 (1.00–1.23)	4246	1.09 (0.98–1.21)
1–5	1940	0.92 (0.79–1.08)	1940	0.96 (0.82–1.12)
6–45	2306	1.26 (1.11–1.43)	2306	1.20 (1.05–1.36)
Blue–collar	14 237			
0	9198	1	9198	1
1–45	5039	1.12 (1.03–1.21)	5039	1.04 (0.95–1.13)
1–5	2047	0.97 (0.86–1.10)	2047	0.95 (0.84–1.08)
6–45	2992	1.22 (1.11–1.34)	2992	1.09 (0.99–1.21)

a Crude model.

b Adjusted for sex, age, education, coffee consumption, smoking, body mass index, severity of disease at baseline, alcohol consumption and leisure-time physical activity. Only individuals with complete data were included.

When gender was added to the analysis, male WCW showed a significant association with mortality for 1–45 years of night work and, particularly, for exposure of 6–45 years (table 4). The association for BCW was not significant.

**Table 4:** Cox proportional crude and adjusted hazard ratios (HR) with 95% confidence intervals (CI) for the association between night work and mortality among women and men stratified by occupational groups.

Years with night shifts	N	Model 1 ^a^ HR (95% CI)	N	Model 2 ^b^ HR (95% CI)
Women				
White-collar	8714			
0 years	6945	1	6945	1
1–45	1769	0.94 (0.79–1.11)	1769	0.95 (0.80–1.13)
1–5	973	0.82 (0.65–1.04)	973	0.85 (0.67–1.08)
6–45	796	1.08 (0.86–1.36)	796	1.06 (0.84–1.34)
Blue-collar	7076			
0	5131	1	5131	1
1–45	1945	0.94 (0.81–1.09)	1945	0.99 (0.85–1.14)
1–5	967	0.83 (0.68–1.01)	967	0.89 (0.73–1.09)
6–45	978	1.05 (0.88–1.26)	978	1.08 (0.90–1.30)
Men				
White-collar	7631			
0	5154	1	5154	1
1–45	2477	1.14 (1.00–1.31)	2477	1.21 (1.06–1.38)
1–5	967	0.98 (0.80–1.20)	967	1.07 (0.87–1.31)
6–45	1510	1.24 (1.07–1.45)	1510	1.28 (1.10–1.50)
Blue-collar	7161			
0	4067	1	4067	1
1–45	3094	1.09 (0.98–1.21)	3094	1.07 (0.96–1.19)
1–5	1080	1.01 (0.87–1.18)	1080	0.99 (0.85–1.15)
6–45	2014	1.14 (1.01–1.28)	2014	1.11 (0.99–1.25)

a Crude model.

b Adjusted for age, education, coffee consumption, smoking, body mass index, severity of disease at baseline, alcohol consumption and leisure-time physical activity. Only individuals with complete data were included.

The group of male WCW night shift workers was made up of managers (18% / 23%) in only day working male (WCW), professionals (MD, teachers, academics, lawyers, psychologists, etc) (28% /0%), college educated (engineers, pilots, nurses, police) (38% / 36%), office employees (11% / 10%), military (3% / 1%). No differences were significant (Chi^2^). The individual occupations in which night work dominated with >2/3 of the occupation, were police (6.2% of all male WCW), pilots (4.8%), MDs (7.8%), and nurses (1.8%).

In the subsample of discordant twin pairs (table 5), the estimates were adjusted for familial factors as well as sex and age. Significant HR were found for being exposed to night work >5 years. However, after further adjusting for the covariates also used in the analyses of the whole cohort (as in the previous tables), no significant results were seen.

**Table 5:** Cox proportional crude and adjusted hazard ratios (HR) with 95% confidence intervals (95% CI) for the association between night work and mortality among discordant twin pairs (N=2074).

	N	Model 1 ^a^ HR (95% CI)	N	Model 2 ^b^ HR (95% CI)
All	2074			
0	1453	1	1453	1
1–45	621	1.09 (0.95–1.24)	621	1.03 (0.90–1.17)
1–5	242	1.05 (0.86–1.27)	242	1.02 (0.84–1.24)
6–45	379	1.11 (0.95–1.30)	379	1.03 (0.88–1.21)
Monozygotic	1576			
0	1111	1	725	1
1–45	465	1.00 (0.86–1.17)	339	1.05 (0.87–1.26)
1–5	184	0.95 (0.75–1.19)	139	0.97 (0.75–1.27)
6–45	281	1.04 (0.87–1.25)	200	1.10 (0.88–1.38)
Dizygotic	2959			
0	2103	1	1409	1
1–45	856	1.09 (0.97–1.22)	606	1.03 (0.89–1.18)
1–5	341	0.98 (0.83–1.16)	241	0.92 (0.74–1.13)
6–45	515	1.16 (1.01–1.33)	365	1.10 (0.93–1.29)

a Adjusted for sex and age as well as for familial factors (ie, genetics and early environment).

b Additionally adjusted for education, coffee consumption, smoking, body mass index, severity of disease at the baseline, and white/blue-collar occupational group, alcohol consumption and leisure-time physical activity. Only individuals with complete data have been included.

All analyses above were repeated with individuals with missing data retained. This only changed the results marginally.

## Discussion

The results showed that 1–45 years of night work was associated with higher risk for all-cause mortality and mortality in circulatory diseases, compared to those not exposed to night work. The effect was stronger among those with longer exposure. The effect was present for both white and blue-collar workers for those with longer exposure. When also gender was considered, a significant effect was present among male WCW only, and particularly among those with longer exposure. Familial factors and gender seem to be of less importance for the associations studied.

The significant overall effect of 1–45 years of exposure to night shifts was relatively modest. As indicated in the introduction, very few similar studies are available, and the results vary. A recent meta-analysis did not find any significant effects of night work (17). Neither did a recent study of a representative sample of 150 000 individuals (18). However, the (non-significant) risk ratio in that study was 1.07, equal to the HR of 1.07 in the present study. Furthermore, the former study did not adjust for life style factors (smoking, alcohol intake, physical activity, BMI, etc) and this may have attenuated the results.

The present study also considered duration of exposure and the results indicate that longer exposure (>5 years) is associated with mortality (as is 14–45 years). Apart from this observation, there was no clear dose–response pattern. Thus, the present data suggests a threshold association with a sudden increase in risk after some years of exposure but no accumulation. This is quite unusual in epidemiological studies. The reason for the lack of influence of amount of exposure is not clear, and other studies of night shifts and mortality have not taken duration of exposure into account. However, a similar threshold has been observed for exposure to night work and breast cancer in the same cohort (25). We have no explanation for the apparent threshold effect, but one possibility is the tendency to leave shift work occupations over time (26). The reasons for poor tolerance for shift work seem to be related to fatigue (27). This turnover may prevent serious diseases or mortality to surface in the short run but may eventually result in an accumulation negative effects in those who have remained in night shift work for a long time. This is very speculative, but should be possible to investigate.

The increased mortality due to cardiovascular disease agrees with previous work on cardiovascular morbidity (28, 29). The increased mortality in cancer only occurred for long-term exposure and cancer morbidity, but this is in line with the classification of night shift work as probably cancerogenic in humans by the International Agency for Research on Cancer (30) .

The finding of a significant effect of night work in WCW agrees with the previous finding (15). The latter and the present study contained representative samples of the population, whereas the other available studies have focused on specific groups, like nurses, who are WCW, and found an increased risk (13, 14). Studies of industrial shift workers (BCW) have failed to find any increased risk (12, 31). However, the nurses and the industrial workers are very different in terms of gender, and it is not possible to use results from those studies as support for an association between night work and mortality being stronger among WCW.

The finding of an increased mortality among male WCW only, contrasts with our previous finding of an increased mortality in female WCW (15). The latter study, however, included lower WCW in the BCW group and also included evening work and other non-night work schedules. It is, therefore, not directly comparable to the present cohort. It should be emphasized that increased, but non-significant, HR were seen for longer exposure also in female WCW, as well as in male and female BCW. The reason for the significantly higher mortality for male WCW in the present study is not clear. A number of possible confounders were adjusted for, including alcohol consumption and smoking, while physical workload was handled through the stratification on WCW and BCW. Furthermore, the distribution of major occupational groups did not differ between day and night shift groups among male WCW. Thus, stress should not be an issue, even if we have no possibility to test this possibility. However, it is possible that shift schedules in male WCW might be more demanding than in other shift work groups, as for example, for pilots (time zone shifts), MD (long on-call shifts), and nurses (frequent quick returns, short rests periods). Unfortunately, the present study does not contain information on shift characteristics, and – to the best of our knowledge – there are no available studies of the health burden of different types of night shift schedules in relation to occupational groups. This seems to be an important question for future research.

The association between longer night shift experience and mortality in male BCW was borderline significant, but supports the notion of the link between night shifts and mortality being present mainly in males. Again, we suggest that the characteristics of the shift system may be part of the explanation, but new research focused on these issues is needed.

The lack of hereditary effect somewhat weakens the argument for an effect of night shift work on mortality. Twin comparisons constitute a strong design for interpretations of causality. However, the demonstrated effect for the whole group was small and it is possible that the proportion of discordant twin pairs may have been too small to detect effects.

There are no studies of the pathophysiology behind the link between night work and mortality, but with regard to night work and *disease*, it has been suggested that the alternation between day and night shifts causes desynchronization of circadian rhythms, and this in turn causes ill health (32). Yet, evidence only derives from animals in simulated night work (33). It is also possible that sleep loss that occurs in connection with night and morning shifts (34) may play a role, since short sleep is associated with mortality (35). However, short sleep here refers to habitual such sleep, not the variation between days of short as long sleep, as found in shift work. Also, alcohol consumption and smoking habits have been suggested as causes of health problems in shift workers (36). The overall impression is that we lack information on the pathophysiology of night work and mortality or health.

The present study has several limitations. A major one is that exposure is only measured once and that we have no information on when exposure occurred. Furthermore, the amount of exposure was based on subjective reports, but objective data on night work exposure are rare in large representative population-based studies, with some exceptions (37). The amount of night work exposure was set at least one night per month. This would have diluted the effects of frequent night work. Probably also factors like frequency of night shifts, shift duration and quick returns (short time between shifts), sleep, age when entering or leaving shift work should be included in studies of night shifts and mortality. In view of this, the present results are restricted to the *duration of exposure* of night shifts, not night shift work *per se*.

The strengths of the study are its size, the long follow-up, the possibility to investigate the influence of familial factors on the associations, and the virtually complete information of mortality through national registers.

In conclusion, the present study has shown that accumulated exposure to night shifts is associated with increased mortality among male WCW, possibly also male BCW, and the effect is increased for exposures >5 years. Also cause-specific mortality with cardiovascular diagnoses was increased, possibly also with cancer, but only after long-term exposure. It should be emphasized that the present results only pertain to the duration of the exposure to night shifts, not night shift work per se (which would include, density of night shifts and other characteristics). The results should be interpreted with caution because of the unexplained absence of effects of accumulated exposure.

## References

[1] Akerstedt T, Kecklund G (2017). What work schedule characteristics constitute a problem to the individual?A representative study of Swedish shift workers. Appl Ergon.

[2] Cheng M, He H, Wang D, Xu L, Wang B, Ho KM (2019). Shift work and ischaemic heart disease:meta-analysis and dose-response relationship. Occup Med (Lond).

[3] Knutsson A, Kempe A (2014). Shift work and diabetes--a systematic review. Chronobiol Int.

[4] Gan Y, Yang C, Tong X, Sun H, Cong Y, Yin X (2015). Shift work and diabetes mellitus:a meta-analysis of observational studies. Occup Environ Med.

[5] Wagstaff AS, Sigstad Lie JA (2011). Shift and night work and long working hours--a systematic review of safety implications. Scand J Work Environ Health.

[6] Hansen J (2017). Night Shift Work and Risk of Breast Cancer. Curr Environ Health Rep.

[7] Hedström AK, Åkerstedt T, Olsson T, Alfredsson L (2015). Shift work influences multiple sclerosis risk. Mult Scler.

[8] Hedström AK, Åkerstedt T, Klareskog L, Alfredsson L (2017). Relationship between shift work and the onset of rheumatoid arthritis. RMD Open.

[9] Thiis-Evensen E (1949). Skiftarbeid og helse. [Shift work and health] Porsgrunn:Andreas Jakobsens Boktrykkeri.

[10] Taylor PJ, Pocock SJ (1972). Mortality of shift and day workers 1956-68. Br J Ind Med.

[11] Knutsson A, Hammar N, Karlsson B (2004). Shift workers'mortality scrutinized. Chronobiol Int.

[12] Karlsson B, Alfredsson L, Knutsson A, Andersson E, Torén K (2005). Total mortality and cause-specific mortality of Swedish shift- and dayworkers in the pulp and paper industry in 1952-2001. Scand J Work Environ Health.

[13] Gu F, Han J, Laden F, Pan A, Caporaso NE, Stampfer MJ (2015). Total and cause-specific mortality of U.S. nurses working rotating night shifts. Am J Prev Med.

[14] Jørgensen JT, Karlsen S, Stayner L, Andersen J, Andersen ZJ (2017). Shift work and overall and cause-specific mortality in the Danish nurse cohort. Scand J Work Environ Health.

[15] Åkerstedt T, Kecklund G, Johansson SE (2004). Shift work and mortality. Chronobiol Int.

[16] Nätti J, Anttila T, Oinas T, Mustosmäki A (2012). Night work and mortality:prospective study among Finnish employees over the time span 1984 to 2008. Chronobiol Int.

[17] Lin X, Chen W, Wei F, Ying M, Wei W, Xie X (2015). Night-shift work increases morbidity of breast cancer and all-cause mortality:a meta-analysis of 16 prospective cohort studies. Sleep Med.

[18] Hannerz H, Soll-Johanning H, Larsen AD, Garde AH (2019). Night-time work and all-cause mortality in the general working population of Denmark. Int Arch Occup Environ Health.

[19] Bøggild H, Knutsson A (1999). Shift work, risk factors and cardiovascular disease. Scand J Work Environ Health.

[20] Toomey R, Panizzon MS, Kremen WS, Franz CE, Lyons MJ (2015). A twin-study of genetic contributions to morningness-eveningness and depression. Chronobiol Int.

[21] Åkerstedt T, Narusyte J, Alexanderson K, Svedberg P (2017). Sleep Duration, Mortality, and Heredity-A Prospective Twin Study. Sleep (Basel).

[22] Lichtenstein P, De Faire U, Floderus B, Svartengren M, Svedberg P, Pedersen NL (2002). The Swedish Twin Registry:a unique resource for clinical, epidemiological and genetic studies. J Intern Med.

[23] Svedberg P, Bardage C, Sandin S, Pedersen NL (2006). A prospective study of health, life-style and psychosocial predictors of self-rated health. Eur J Epidemiol.

[24] Ropponen A, Narusyte J, Alexanderson K, Svedberg P (2011). Stability and change in health behaviours as predictors for disability pension:a prospective cohort study of Swedish twins. BMC Public Health.

[25] Åkerstedt T, Knutsson A, Narusyte J, Svedberg P, Kecklund G, Alexanderson K (2015). Night work and breast cancer in women:a Swedish cohort study. BMJ Open.

[26] Knutsson A, Åkerstedt T (1992). The healthy-worker effect:self-selection among Swedish shift workers. Work Stress.

[27] Axelsson J, Åkerstedt T, Kecklund G, Lowden A (2004). Tolerance to shift work-how does it relate to sleep and wakefulness?. Int Arch Occup Environ Health.

[28] Knutsson A, Åkerstedt T, Jonsson BG, Orth-Gomér K (1986). Increased risk of ischaemic heart disease in shift workers. Lancet.

[29] Frost P, Kolstad HA, Bonde JP (2009). Shift work and the risk of ischemic heart disease - a systematic review of the epidemiologic evidence. Scand J Work Environ Health.

[30] IARC Monographs. Vol 124 group. Carcinogenicity of night shift work (2019). Lancet Oncol.

[31] Bøggild H, Suadicani P, Hein HO, Gyntelberg F (1999). Shift work, social class, and ischaemic heart disease in middle aged and elderly men a 22 year follow up in the Copenhagen Male Study. Occup Environ Med.

[32] Leger D, Esquirol Y, Gronfier C, Metlaine A (2018). Groupe consensus chronobiologie et sommeil de la Societe francaise de recherche et medecine du sommeil. Le travail postéet de nuit et ses conséquences sur la santé:état des lieux et recommandations [Shift-workers and night-workers'health consequences:State of art and recommendations]. Presse Med.

[33] Park N, Cheon S, Son GH, Cho S, Kim K (2012). Chronic circadian disturbance by a shortened light-dark cycle increases mortality. Neurobiol Aging.

[34] Åkerstedt T, Kecklund G (2011). Shift work, severe sleepiness and safety. Ind Health.

[35] Cappuccio FP, D'Elia L, Strazzullo P, Miller MA (2010). Sleep duration and all-cause mortality:a systematic review and meta-analysis of prospective studies. Sleep.

[36] Knutsson A (2004). Methodological aspects of shift-work research. Chronobiol Int.

[37] Cordina-Duverger E, Menegaux F, Popa A, Rabstein S, Harth V, Pesch B (2018). Night shift work and breast cancer:a pooled analysis of population-based case-control studies with complete work history. Eur J Epidemiol.

